# Shwachman-Diamond syndrome associated with rod-cone dystrophy

**DOI:** 10.1097/ICB.0000000000001568

**Published:** 2025-03-01

**Authors:** Jingwen Zhang, Thales A. C. de Guimaraes, Dorothy Thompson, Michel Michaelides

**Affiliations:** 1 UCL Institute of Ophthalmology, University College London, London, UK; 2 Moorfields Eye Hospital, London, UK; 3 Great Ormond Street Hospital, London, UK

**Keywords:** ophthalmic genetics, rod-cone dystrophy, Shwachman-Diamond syndrome

## Abstract

**Purpose:**

To report a patient with Shwachman-Diamond syndrome and concomitant rod-cone dystrophy who underwent bone marrow transplantation.

**Methods:**

Retrospective single case report.

**Results:**

A female patient with Shwachman-Diamond syndrome was referred to a tertiary hospital to investigate possible pigmentary retinopathy at the age of 16. She described poor night vision and was found to have reduced visual acuity (6/20 right, 6/38 left). Over the ten-year follow-up period, her visual acuity remained relatively stable with no new visual symptoms. Optical coherence tomography revealed progressive, diffuse outer retinal thinning with disruption of the ellipsoid zone, which initially was relatively preserved subfoveally. Fundus autofluorescence images revealed generalised areas of hypoautofluorescence beyond the vascular arcades and a perimacular ring of increased autofluorescence. The flash electroretinogram was in keeping with a severe rod-cone dystrophy. The pattern visual evoked potential was abnormal but detectable indicating macular pathway dysfunction, suggesting encroachment into central macular regions but with some functional preservation.

**Conclusions:**

We report a patient with Shwachman-Diamond syndrome with severe early-onset rod-cone dystrophy noted at the age of 16. Slow anatomical progression has been observed over the subsequent ten years, with relative functional macular preservation to support a visual acuity of 6/36 in both eyes.

## Introduction

Shwachman-Diamond syndrome (SDS) is a rare multisystemic disorder. It is genetically heterogeneous; most commonly inherited in an autosomal recessive fashion due to pathogenic biallelic variants in the *DNAJC21* (OMIM #617048), *EFL1* (OMIM #617538) or *SBDS* (OMIM #607444) genes. It can also be inherited in an autosomal dominant fashion due to heterozygous pathogenic variants in the *SRP54* (OMIM #604857) gene.^1^

More than 90% of cases are due to *SBDS* (OMIM #607444). *SBDS* encodes a highly conserved protein involved in ribosomal functioning.^1^ It was found that the *SBDS* protein releases the eukaryotic initiation factor 6 from the 60S ribosomal subunit by coupling the energy liberated from the GTP hydrolysis by elongation factor-like GTPase 1 (*EFL1*); this way, *SBDS* allows the assembly of the 80S subunit, playing an essential role in ribosome biogenesis.^2^

In almost all affected children, persistent or intermittent neutropenia is a common presenting finding, often before the diagnosis of SDS is made. Short stature and recurrent infections are common. The classical findings necessary to establish the diagnosis of SDS, a ribosomopathy, are exocrine pancreatic dysfunction and bone marrow failure.^1^ Phenotypes may vary considerably between individuals. The constellation of findings include bone marrow malfunction with susceptibility to developing myelodysplastic syndromes, aplastic anaemia, acute myeloid leukaemia, exocrine pancreatic insufficiency, immunodeficiency, skeletal abnormalities, intellectual disabilities, sensorineural hearing loss and liver, heart, skin, endocrine and teeth complications.^3^ Additionally, ophthalmic features have been observed in some affected individuals, including rare cases of retinitis pigmentosa and retinal dystrophy.^4,5^

Herein, we report the ten-year follow-up of a rare case of SDS with rod-cone dystrophy.

## Case report

The patient is now a 27-year-old female with SDS. Her medical history is complex. Early in her life, she received bone marrow transplants (BMT) due to bone marrow dysfunction. At one year of age, she received her first BMT, which later failed and required a second BMT at the age of three. The second BMT was successful and achieved 100% donor haematopoiesis. Prior to her referral to Ophthalmology, the patient was under Endocrinology for exocrine pancreatic insufficiency, severe insulin resistance and diabetes mellitus, as part of her systemic syndrome. She was also diagnosed with sensorineural hearing loss and learning difficulties, as well as amelogenesis imperfecta.

Genetic testing was not performed due to the history of two allogeneic BMTs. Her family history is not significant for retinal diseases, but her mother was found to be heterozygous for a previously reported pathogenic variant in *SBDS*, c.183_184delinsCT (p.Lys62Ter), which introduces an in-frame stop codon. Her father was negative for the pathologic variant and her parents are unrelated. She also has two unaffected female siblings.

She was referred to a tertiary Ophthalmology service to investigate possible pigmentary changes in the retinal periphery at the age of 16. At referral, her visual acuity was 6/20 (LogMAR 0.52) in the right eye (OD) and 6/38 (LogMAR 0.8) in the left eye (OS) at two meters. She was able to read the N4.5 near vision chart at 0.45 meters with both eyes open. She was found to have low hyperopia. Left exotropia and low amplitude, moderate frequency nystagmus were noted. She was diagnosed with amblyopia in the left eye.

**Figure 1:**
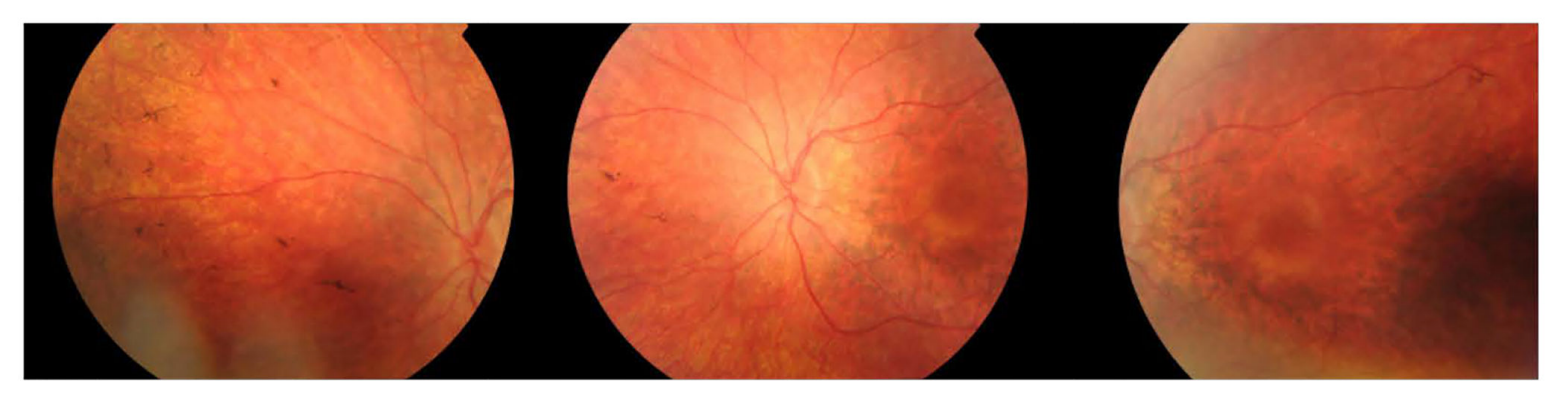
Colour fundus images of the left eye showed a relatively crowded optic disc, vessel attenuation and diffuse RPE mottling with a central macular island of preservation.

Slit lamp examination revealed clear media. Fundus examination showed crowded optic discs, diffuse retinal pigment epithelium (RPE) pigmentary changes and bilateral RPE pigment migration peripherally with a central macular island of relative preservation. The fundus images are shown in Figure 1.

**Figure 2:**
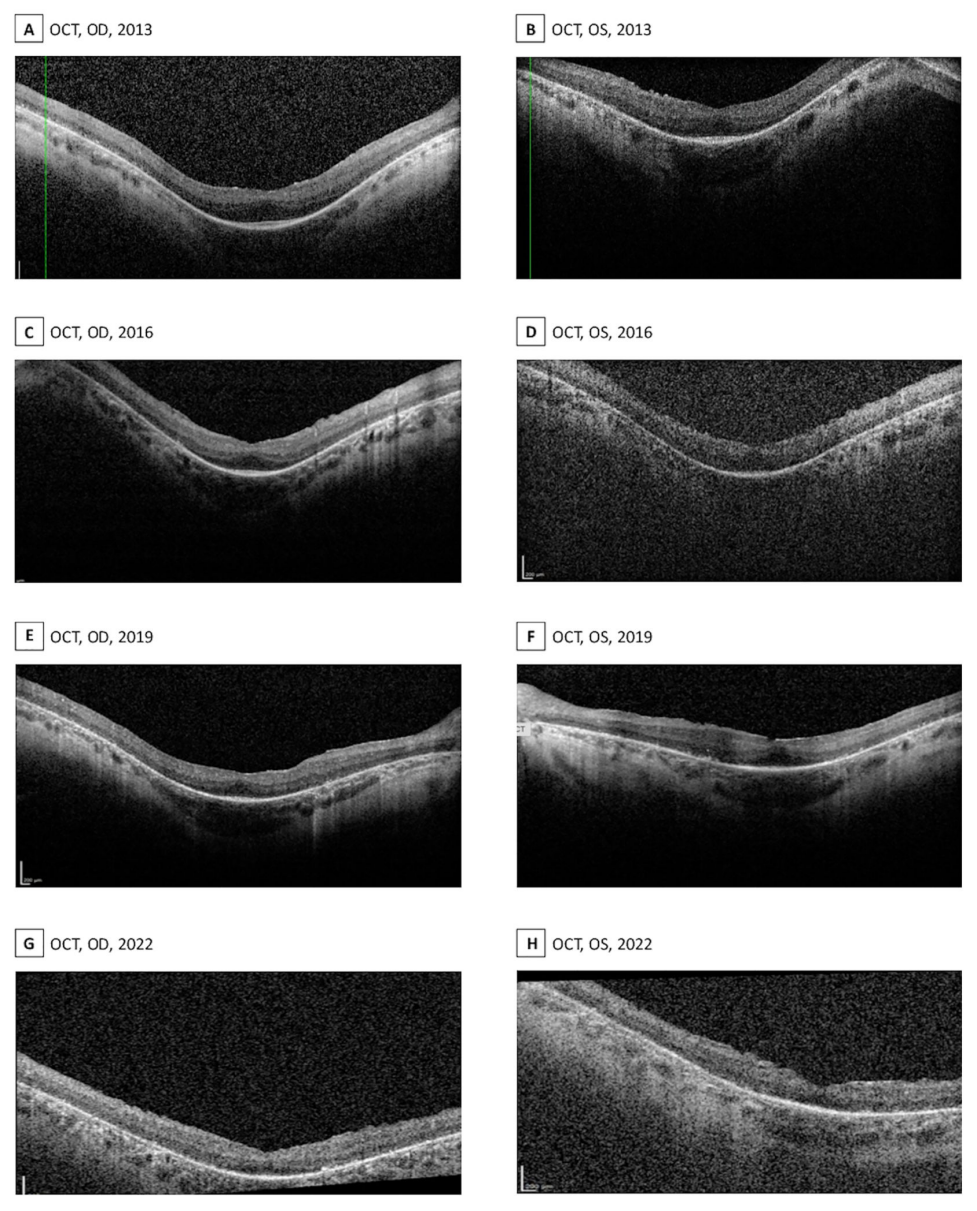
Initial OCTs of the right eye (A) and left eye (B) in 2013 showed diffuse outer retinal thinning with disruption to the EZ, which was somewhat preserved subfoveally. Over time, as shown by the OCTs from 2016 to 2022, images (C) to (H), there was progressive outer retinal thinning and further disruption to the EZ in both eyes, indicating that the patient is in a later stage of the disease based on imaging.

**Figure 3:**
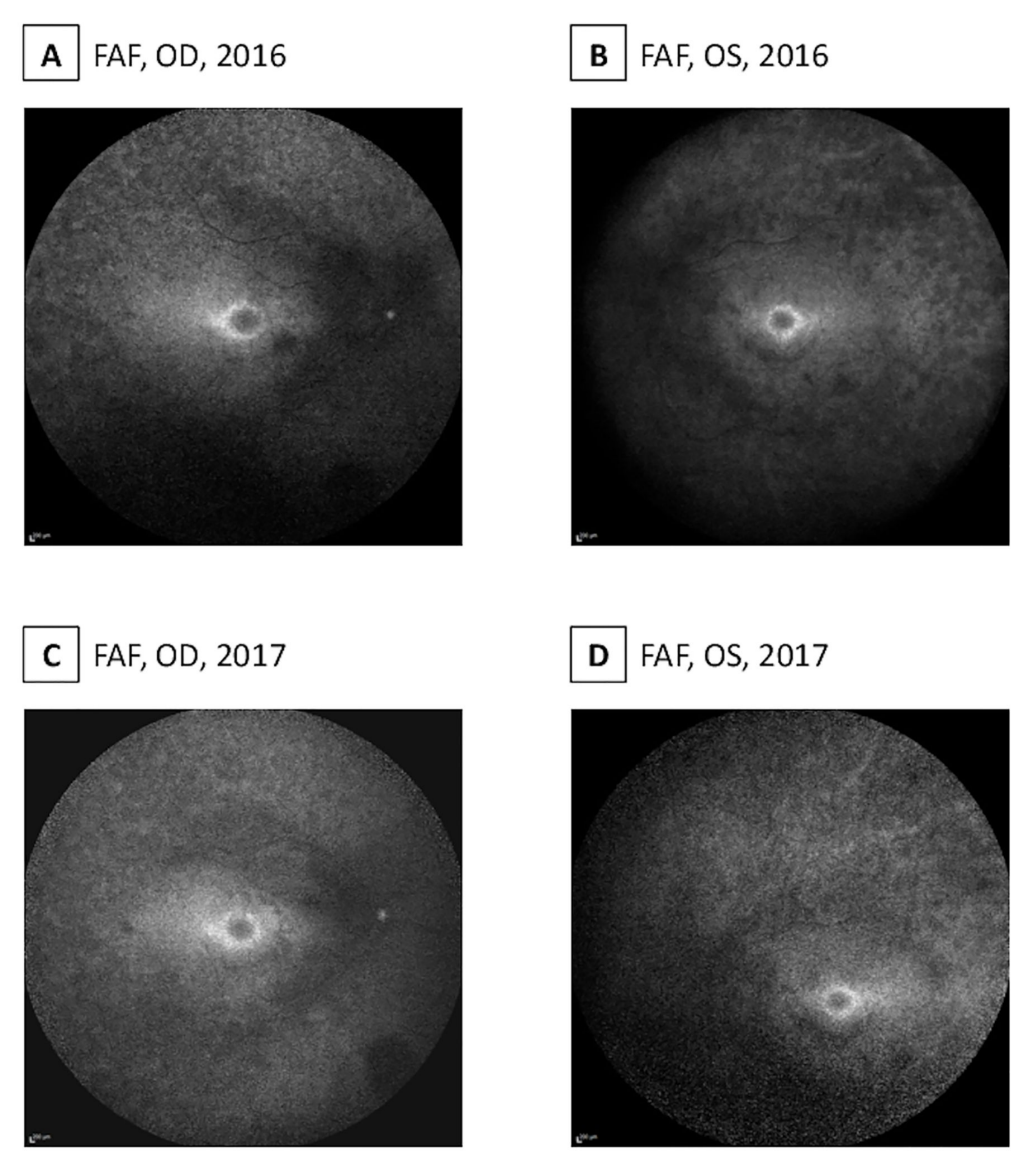
FAFs showed diffuse RPE mottling with areas of hypoautofluorescence outside of the vascular arcades and a perimacular hyperautofluorescent ring.

Optical coherence tomography (OCT) revealed diffuse outer retinal thinning with loss of peripheral ellipsoid zone (EZ) and relative preservation centrally and paracentral atrophy (Fig. 2 A and B). Fundus autofluorescence (FAF) was obtained with difficulty due to the nystagmus, but a hyperautofluorescent perimacular ring was evident. Similarly, there is a diffusely reduced autofluorescent signal throughout the retina with 360-degree hypoautofluorescent patches from vascular arcades to mid-periphery (Fig. 3).

**Figure 4:**
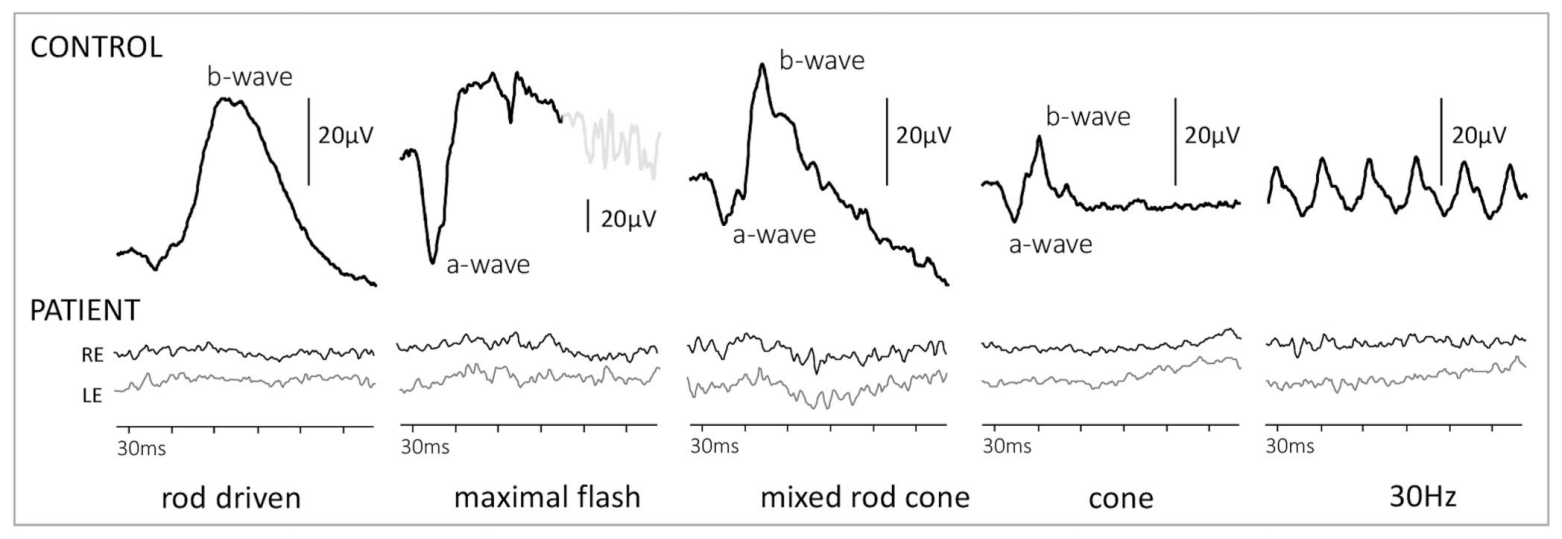
Flash ERGs from the patient’s right and left eye at 16 years of age compared to exemplary waveforms from healthy control above to different flash stimuli. The a-wave arises mainly from photoreceptors and b-wave from bipolar cells. The patient’s ERGs, a- and hence b-waves, are not detectable which indicates severe, generalised, bilateral retinal rod-cone dystrophy. (Marmoy OR, Moinuddin M, Thompson DA. An alternative electroretinography protocol for children: a study of diagnostic agreement and accuracy relative to ISCEV standard electroretinograms. Acta Ophthalmol. 2022 May;100(3):322-330. doi: 10.1111/aos.14938. Epub 2021 Jun 14. PMID: 34126657.)

**Figure 5:**
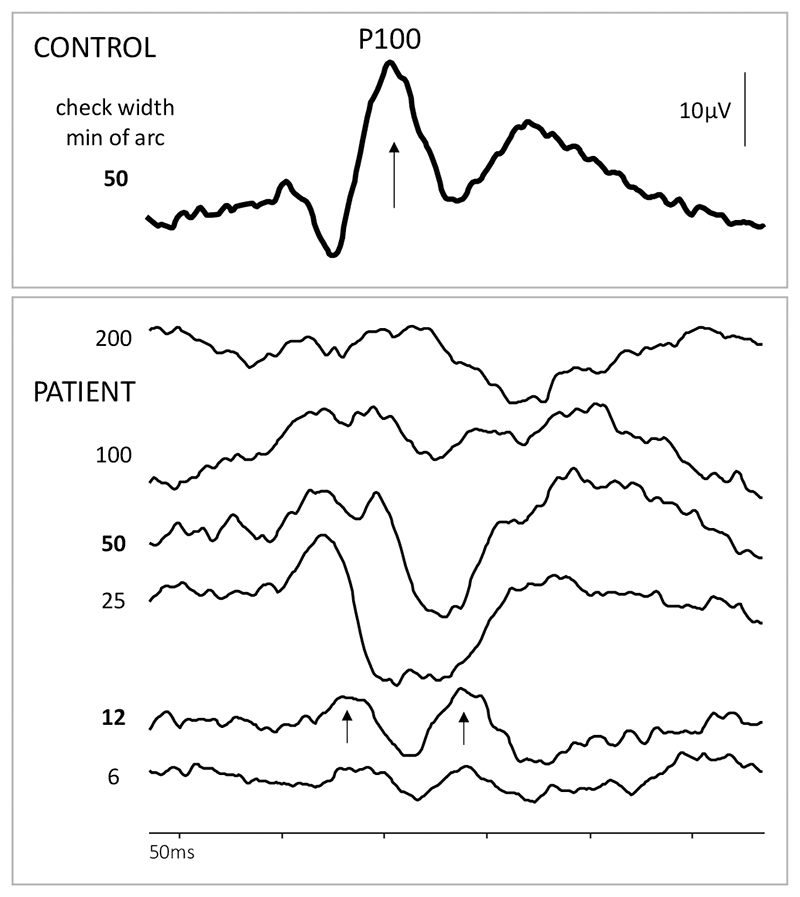
An exemplary pattern reversal VEP (PrVEP) from a healthy control to ISCEV standard large check width 50’. The main positive peak P100 is arrowed. PrVEPs from the patient at 16 years of age are displayed below to a range of check widths presented with both eyes open. ISCEV standard check widths are in bold (50 and 12 min of arc). The patient’s PrVEPs have an atypical waveshape; P100 is not recognized – rather, there are two peaks, arrowed. This indicates bilateral macular pathway dysfunction. A detectable PrVEP nonetheless indicates some macular pathway function and presumed retinal macular function. (Odom JV, Bach M, Brigell M, Holder GE, McCulloch DL, Mizota A, Tormene AP; International Society for Clinical Electrophysiology of Vision. ISCEV standard for clinical visual evoked potentials: (2016 update). Doc Ophthalmol. 2016 Aug;133(1):1-9. doi: 10.1007/s10633-016-9553-y. Epub 2016 Jul 21. PMID: 27443562.)

The flash electroretinogram (ERG) showed severe rod-cone dystrophy (Fig. 4). The pattern-reversal visual evoked potentials (prVEP) showed some preservation of macular pathway function, but atypical prVEP waveforms indicated some encroachment into the central retina (Fig. 5).

From 2012 to 2022, her visual acuity in the left eye remained stable (6/38-6/36), with a change from 6/20 to 6/36 in the right eye, with no new symptoms. However, there was progressive outer retinal thinning, evident on serial OCTs (Fig. 2 C to H), as well as a qualitative increase in the hyperautofluorescent ring area, as seen in the FAF (Fig. 3). She uses 50% LTF sunglasses, a brightfield magnifier and a small 4x pocket magnifier for fine details.

## Discussion

We have documented severe rod-cone dystrophy over a ten-year period in a patient with SDS. Currently, the pathogenesis of retinal changes in SDS is not well understood with only a few cases reported,^4,5^ making it more challenging to definitively establish the association between SDS and retinal dystrophy. The aim of the case report is to report a possible association between SDS and rod-cone degeneration, which may be helpful for ophthalmologists encountering similar cases.

Genetic testing was not performed in this patient. Following pre-transplant myeloablation in the recipient, donor bone marrow haematopoietic stem cells will proliferate and reconstitute the immune system in the recipient. It is well-accepted that the recipient’s blood cells may be replaced by the donor genotype. But the donor stem cells can also transdifferentiate or dedifferentiate into tissue-specific cells, including buccal and epithelial cells, which have been considered alternatives for genetic testing in these patients.^6^ Our patient has gone through two BMTs with stem cells from different donors. Thus, due to the challenges in DNA isolation, genetic testing was not performed for this patient.

In a case series of 21 clinically diagnosed SDS individuals, four patients, from age one to age 16, were described with retinal dysfunction. The eldest had reduced visual acuity associated with optic atrophy and retinitis pigmentosa; all others were reported to have normal visual acuity. Two patients, both at one year old, were described as having reduced retinal and choroidal pigment, one of whom had a generalised reduction in ERG amplitude consistent with retinitis pigmentosa. A fourth patient with retinitis pigmentosa aged seven years also showed a deterioration of IQ.^5^ Another case series reported inflammatory eye conditions in three out of five patients with SDS: two patients presented with bilateral blepharoconjunctivitis, and one patient presented with bilateral keratoconjunctivitis, episcleritis, blepharitis, recurrent chalazions and styes.^7^ Bilateral multiple cilioretinal arteries were reported in one patient with *SDBS*-associated SDS who suffered temporary visual disturbances during hospitalisation for a bone marrow biopsy.^8^

Three of eight patients with SDS due to biallelic pathologic variants in *DNAJC21* have been reported with retinal dystrophy. One showed clinical signs of retinal dystrophy at the age of five, with vessel attenuation and peripheral RPE changes outside the arcades, which was confirmed by an ERG at six and ten years old as a progressive severe rod-cone dystrophy. The patient’s OCTs showed disruption to the photoreceptor inner and outer segments outside of the central macula. She also carried an insertion variant in C2orf71, a gene known to cause retinitis pigmentosa type 54. The second patient developed retinal ‘dysplasia’ without pigmentation after a successful BMT at the age of two, noted as having low visual acuity and constricted visual fields. The third had poor vision and retinal dystrophy noted at the age of six. Thus, it was hypothesized that there may be an association between pathologic variants in the *DNAJC21* gene and retinal dystrophy.^4^

It is also worth considering a separate clinical entity – bone marrow transplantation retinopathy: *all SDS children who are reported with retinal dystrophy have had BMT*. Posterior segment complications were reported in 12.8% of patients undergoing BMT in a study, including 3.5% with haemorrhagic complications (intraretinal and/or vitreous haemorrhages), 4.3% with cotton-wool spot development and 2.8% with optic disc oedema.^9^ A case of progressive outer retinal necrosis was reported in a ten-year-old child after undergoing bone marrow transplantation for Hodgkin’s Lymphoma.^10^ However, rod-cone dystrophy has not been reported as a consequence of BMT.

## Conclusions

We report an unusual case of a patient with SDS, possibly associated with *SBDS*, and severe rod-cone retinal dystrophy. The patient’s visual acuity remained relatively stable over the ten-year follow-up period, with mild progressive structural retinal changes. This case highlights the importance of a detailed ophthalmic examination in SDS. More evidence is needed to elucidate the link between SDS and retinal dystrophy.

## Summary statement

Shwachman-Diamond syndrome is a genetically heterogeneous multisystem disorder. We herein report a patient with Shwachman-Diamond syndrome and concomitant rod-cone dystrophy with a follow-up period of ten years.
